# LncRNA RPPH1 acts as a molecular sponge for miR-122 to regulate Wnt1/β-catenin signaling in hepatocellular carcinoma

**DOI:** 10.7150/ijms.68778

**Published:** 2023-01-01

**Authors:** Jian Zhou, Kun Shi, Weifeng Huang, Yuke Zhang, Qingsong Chen, Tong Mou, Zhongjun Wu, Xufu Wei

**Affiliations:** 1 Department of Hepatobiliary Surgery, The People's Hospital of Rongchang District, Chongqing, China.; 2 Department of Hepatobiliary Surgery, The First Affiliated Hospital of Chongqing Medical University, Chongqing, China.; 3 Department of Traumatology, Chongqing University Central Hospital, Chongqing, China.

**Keywords:** lncRNA RPPH1, miR-122, competing endogenous RNA, Wnt signaling, hepatocellular carcinoma.

## Abstract

This study aimed to explore the role of lncRNA RPPH1 in hepatocellular carcinoma. The expression of RPPH1 and miR-122 was determined by Real-time PCR. Cell proliferation and colony formation assays were employed to monitor cell growth *in vitro*. Wound healing and Transwell assays were applied to detect cell migration and invasion. A dual-luciferase reporter assay was used to verify the interaction between RPPH1 and miR-122. The *in vivo* function of RPPH1 was illustrated by xenograft tumor models. The results showed that the expression of RPPH1 was markedly upregulated in human HCC specimens and cell lines compared to normal controls. However, the trend of miR-122 was the opposite. RPPH1 facilitates the proliferation, migration, and invasion of HCC cells and synchronously suppresses cell apoptosis. The dual-luciferase assay confirmed the relationship between RPPH1 and miR-122. Rescue experiments showed that RPPH1 acted as a competing endogenous RNA (ceRNA) by sponging miR-122 in HCC cells. Moreover, RPPH1 positively regulated the expression of Wnt1 and its downstream targets through miR-122. Our study demonstrates for the first time that RPPH1 promotes HCC progression via the miR-122/Wnt1/β-catenin axis, which may represent a valuable therapeutic target for patients with HCC.

## Introduction

Hepatocellular carcinoma (HCC), which accounts for 75%-80% of liver cancers, is one of the most common malignancies worldwide and has high morbidity and mortality rates 1. Additionally, HCC is one of the most devastating cancers in China owing to the high hepatitis B virus (HBV) infection rate, which is a major risk factor for HCC 2. Although researchers have made efforts to identify potential therapeutic targets to improve the diagnostic and treatment standards for HCC, the prognosis for patients with HCC remains unsatisfactory 3. Therefore, further research is needed to establish novel diagnostic and therapeutic targets for HCC.

Long non-coding RNAs (lncRNAs), which are defined as a group of endogenous non-coding RNAs more than 200 bp in length, have been widely reported in studies of cancers, including HCC 4-7. Recent studies that have focused on the regulatory mechanism of lncRNAs have shown that the crosstalk between lncRNAs and mRNAs occurs by competing for shared microRNAs (miRNAs) response elements. In brief, lncRNAs may act as competing endogenous RNAs (ceRNAs) to sponge miRNAs, sequentially modulating the derepression of miRNA targets and imposing an additional layer of downstream mRNAs 8, 9. For example, lncRNA DSCR8 (Down syndrome critical region 8) functions as a molecular sponge for miR-485-5p to promote HCC cells migration and invasion 10. Moreover, lncRNA MCM3AP antisense RNA 1 (MCM3AP-AS1) exerts its promotion effects on HCC cells through the MCM3AP-AS1/miR-194-5p/FOXA1 pathway 11.

Recently, accumulating studies have shown that lncRNA RPPH1, the RNA component of the RNase P ribonucleoprotein, is involved in the progression of many human diseases. For example, RPPH1 promotes colorectal cancer cell metastasis by binding to β-III tubulin (TUBB3) and enhancing M2 macrophage polarization 12. RPPH1 can directly interact with Galectin-3 (Gal-3) to promote the inflammation and proliferation of mesangial cells in diabetic nephropathy 13. Nonetheless, research on the regulatory mechanism of RPPH1 is not sufficiently comprehensive or in-depth.

In this study, we explored the functions and underlying mechanism of RPPH1 in HCC. The proliferation, migration, and invasion of HCC cells were obviously suppressed in RPPH1 knockdown models. Moreover, we found that RPPH1 exerted an enhancing effect on cell viability and metastasis by activating the Wnt1/β-catenin pathway through sponging miR-122. These results suggest that RPPH1 is a valid therapeutic target for HCC.

## Results

### Expression and clinical significance of RPPH1 in HCC

**Figure 1:**
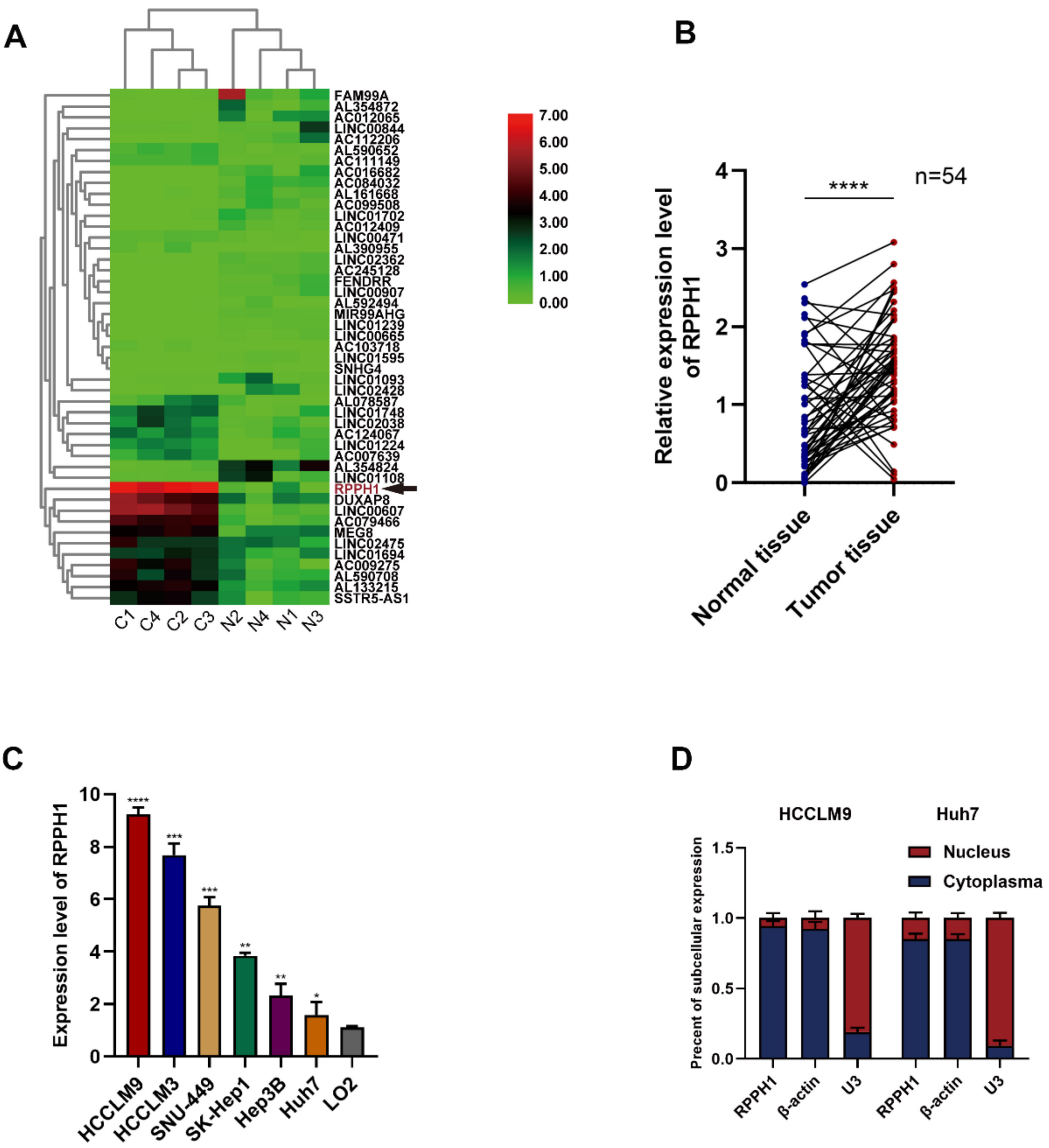
** Expression of RPPH1 in HCC tissues and cell lines. (A)** A heatmap showing that RPPH1 is one of the most abundant differentially expressed lncRNAs in TCGA-LIHC patients. **(B)** qRT-PCR was conducted to detect the expression of RPPH1 in HCC tissues (n = 54). **(C)** Expression of RPPH1 in HCC cell lines and LO2 cells. **(D)** Subcellular RNA fractionation was used for location analysis of RPPH1. Data are presented as the mean ± SD. *p < 0.05, **p < 0.01, ***p < 0.001, ****p < 0.0001.

First, we obtained RPPH1 was one of the most abundant lncRNAs in TCGA-LIHC (Fig. 1A). To investigate the role of RPPH1 in HCC, RPPH1 expression level in 54 paired tissues and cell lines were detected by qRT-PCR. The results showed that the RPPH1 expression level was obviously increased in tumor tissues compared to the corresponding normal tissues (Fig. 1B). Additionally, the level of RPPH1 was significantly increased in different HCC cell lines compared to the normal hepatic cell line LO2 (Fig. 1C). These data indicated that RPPH1 is an oncogene in HCC. RPPH1 expression was highest in HCCLM9 cells and lowest in Huh7 cells among the HCC cell lines assessed. Therefore, the HCCLM9 and Huh7 cell lines were used in the subsequent experiments. Subcellular RNA fractionation was used for location analysis of RPPH1, and the results showed that RPPH1 was abundant in the cytoplasm of HCC cells (Fig. 1D).

### RPPH1 promotes proliferation and inhibits apoptosis of HCC cells

**Figure 2:**
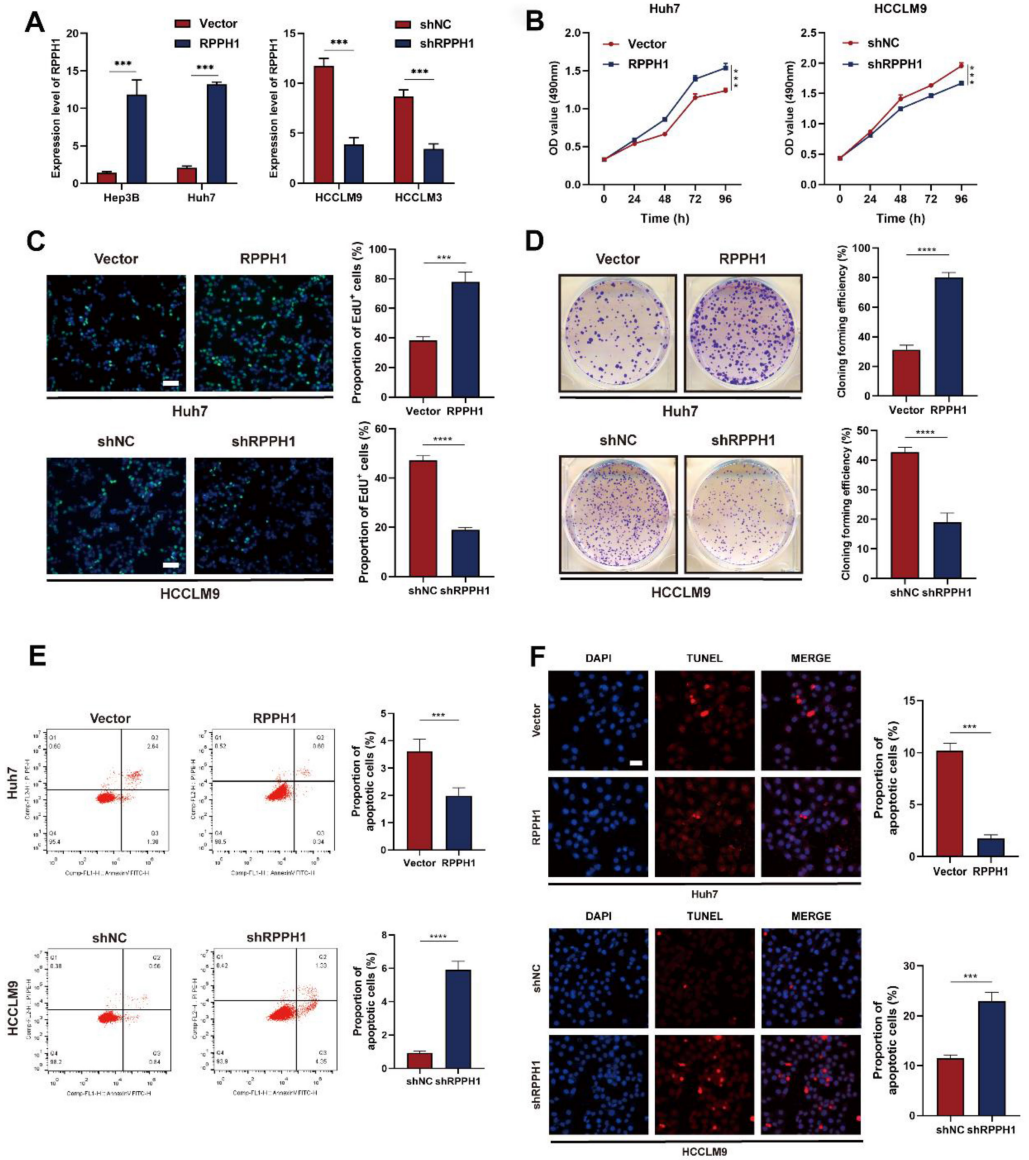
** RPPH1 regulates HCC cell proliferation and apoptosis. (A)** The expression of RPPH1 in HCC cells was measured by qRT-PCR following transfection with RPPH1 or shRPPH1. **(B, C)** MTS and EdU assays showed that RPPH1 promoted the growth of Huh7 cells, while shRPPH1 repressed the growth of HCCLM9 cells.** (D)** Colony formation assays showed that colony-forming efficiency was increased by RPPH1 and decreased by shRPPH1. **(E, F)** Flow cytometry and TUNEL staining showed that RPPH1 reduced, and shRPPH1 induced the apoptosis of HCC cells. Data are presented as the mean ± SD. *p < 0.05, ** *p* < 0.01, ***p < 0.001, ****p < 0.0001.

We decreased RPPH1 expression in HCCLM9 and HCCLM3 cells using pHB-RPPH1 shRNA and increased RPPH1 expression in Huh7 and Hep3B cells using pcDNA3.1-RPPH1 respectively. The transfection efficiency was validated by qRT-PCR (Fig. 2A). Then, MTS, EdU, colony formation, flow cytometry apoptosis, and TUNEL assays were conducted to assess the effects of RPPH1 on the proliferation and apoptosis of HCC cells. The results showed that RPPH1 accelerated the proliferation and repressed apoptosis of Huh7 cells, while shRPPH1 inhibited the proliferation and promoted apoptosis of HCCLM9 cells (Fig. 2B-2F). Therefore, we conclude that RPPH1 facilitates the proliferation and inhibits apoptosis of HCC cells.

### RPPH1 promotes metastasis and growth of HCC cells

**Figure 3:**
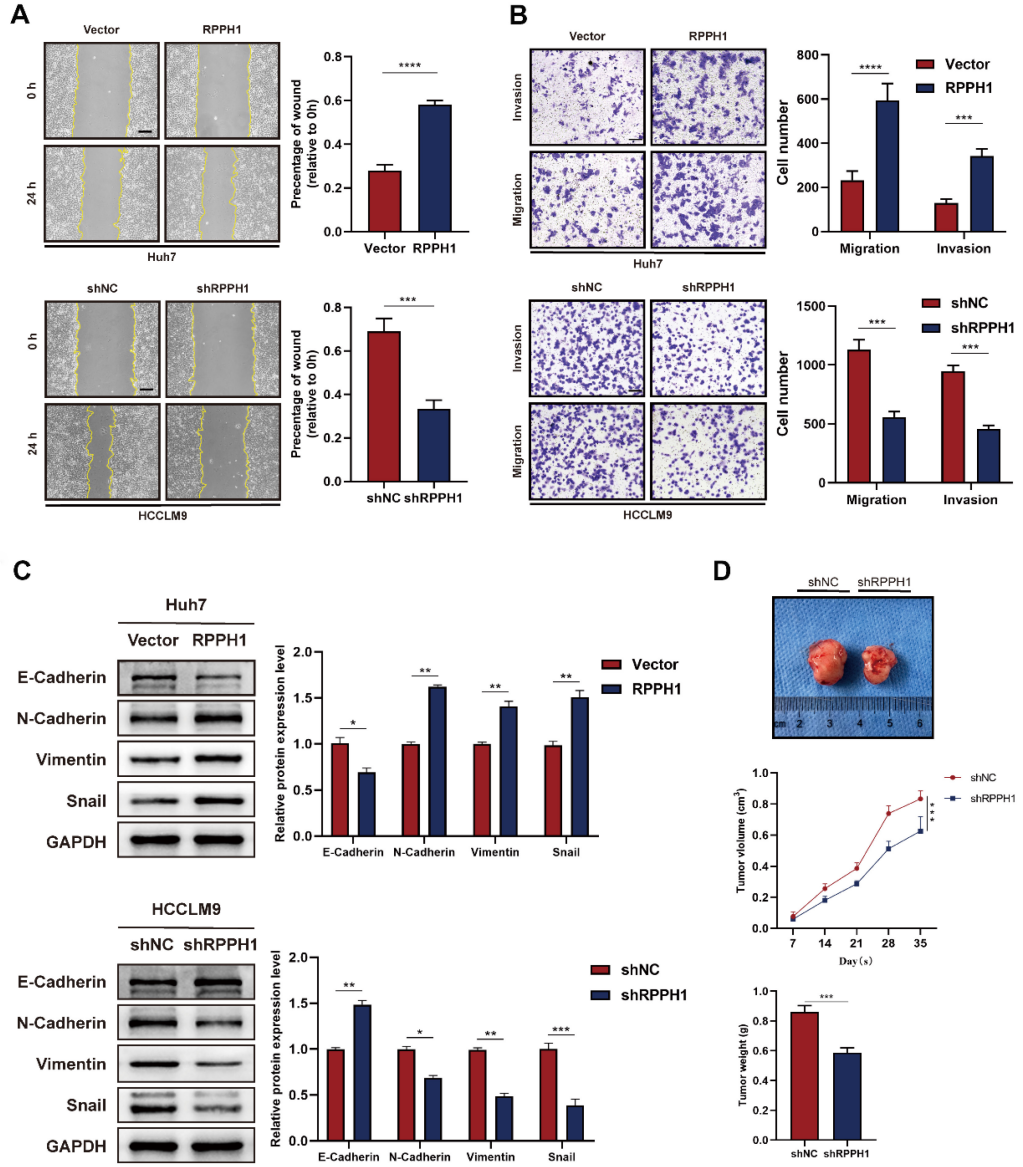
** RPPH1 promotes HCC cell metastasis and tumor growth. (A, B)** Wound healing and transwell assays were conducted to determine the migration and invasion ability of the indicated HCC cells. **(C)** Expression levels of EMT markers E-cadherin, N-cadherin, Vimentin, and Snail in the indicated cells. **(D)** Tumor nodes with shRPPH1 were smaller tumor volume and lighter weight than those with shNC. Data are presented as the mean ± SD. *p < 0.05, **p < 0.01, ***p < 0.001, ****p < 0.0001.

We next explored whether RPPH1 affects the migration and invasion of HCC cells. Wound healing and Transwell assays were performed in HCC cells transfected with the RPPH1 or shRPPH1. Wound healing assay showed that Huh7 cell mobility was strikingly promoted by RPPH1 (Fig. 3A). Transwell assays also showed that RPPH1 increased the number of migrated and invaded cells (Fig. 3B). Since the epithelial-to-mesenchymal transition (EMT) is a crucial factor promoting the migration and invasion of cancer cells, we further investigated whether RPPH1 regulates the EMT of HCC cells. The western blotting data showed that RPPH1 inhibited the expression of the epithelial marker E-cadherin but increased the expression of mesenchymal markers, including N-cadherin, Vimentin, and Snail. shRPPH1 had the opposite effect on HCCLM9 cell metastasis (Fig. 3C). Then, we evaluated the effect of RPPH1 on solid tumor formation in nude mice. We found that there was a distinct reduction in the volume and weight of tumor nodes in the shRPPH1 group compared to those in the control group (Fig. 3D). These results demonstrate that RPPH1 plays a vital tumor-promoting role in HCC progression.

### miR‑122 is a downstream target gene of RPPH1

**Figure 4:**
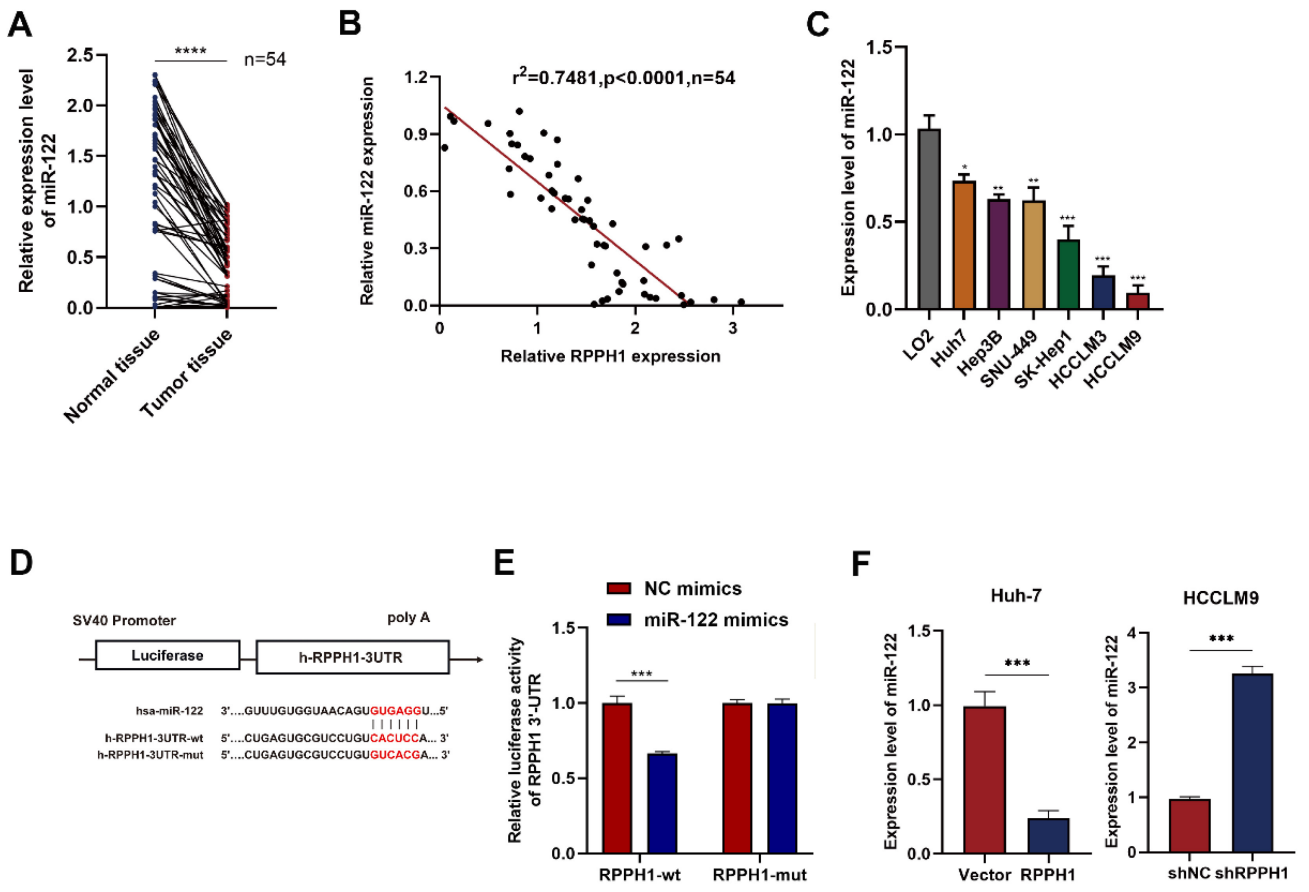
** RPPH1 acts as a sponge for miR-122. (A)** Expression of miR-122 in HCC tissues (n = 54). **(B)** The negative correlation between RPPH1 and miR-122 expression in HCC tissues. **(C)** Expression of miR-122 in LO2 and HCC cells. **(D, E)** The predicted binding area of RPPH1 and miR-122 and dual-luciferase assays showed that miR-122 mimics negatively regulated the luciferase activity of wt RPPH1-3'-UTR. (F) The expression of miR-122 after transfection with RPPH1 or shRPPH1 in HCC cells. Data are presented as the mean ± SD. *p < 0.05, **p < 0.01, ***p < 0.001, ****p < 0.0001.

We next focused on miR-122 and detected its expression in HCC tissues and cell lines by qRT-PCR. The results showed that the RPPH1 expression was significantly impaired in tumor tissues compared to paired normal tissues (Fig. 4A). As expected, a negative correlation between RPPH1 and miR-122 expression was established by Spearman correlation analysis in HCC samples (Fig. 4B). Furthermore, the expression of miR-122 also exhibited an opposite trend to that of RPPH1 in HCC cells (Fig. 4C). The online software miRcode (http://www.mircode.org/) was used to predict the potential target gene of RPPH1. The prediction results showed that RPPH1 has a target binding site for miR-122. The dual-luciferase reporter gene assay in HCCLM9 cells showed that the luciferase activity was notably decreased by co-transfection with the miR-122 mimics and wt RPPH1-3'-UTR, while the miR-122 mimics did not change the luciferase activity of the mut RPPH1-3'-UTR (Fig. 4D, E). We next detected the expression of miR-122 in Huh7 and HCCLM9 cells, which were transfected with RPPH1 and shRPPH1 respectively. The qRT-PCR results showed that RPPH1 ablated the expression of miR-122, while shRPPH1 elevated the expression of miR-122 (Fig. 4F). Taken together, these data suggest that RPPH1 acts as a molecular sponge for miR-122 in HCC cells.

### miR-122 mediates the effects of RPPH1 of HCC cells

**Figure 5:**
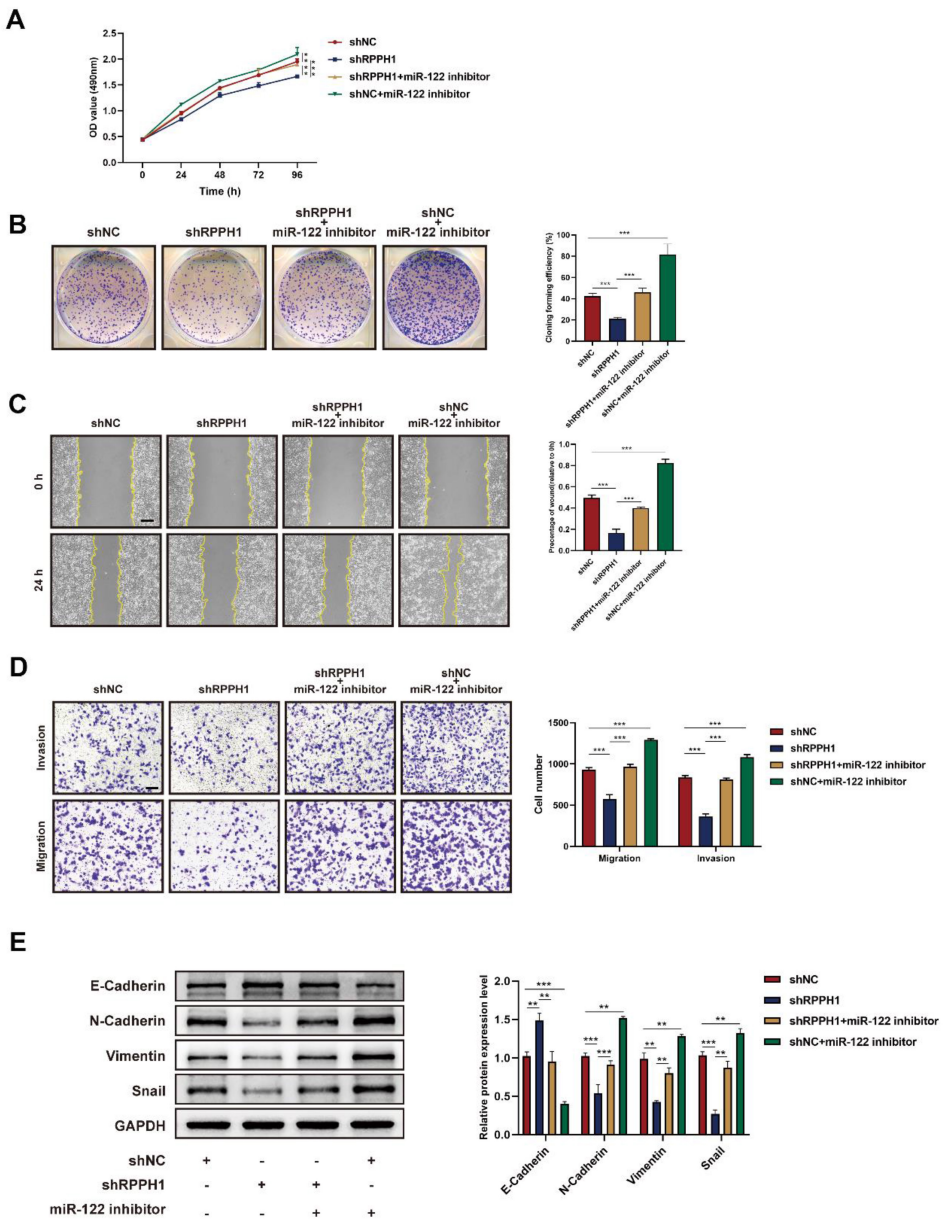
** Functions of RPPH1 are reversed by miR-122. (A, B)** MTS and colony formation assays were used to detect the proliferation ability of HCCLM9 cells. **(C, D)** Wound healing and transwell assays were conducted to determine the migration and invasion ability of HCCLM9 cells. (E) Western blot was used to detect the expression levels of EMT markers E-cadherin, N-cadherin, Vimentin, and Snail in HCCLM9 cells. Data are presented as mean ± SD. *p < 0.05, **p < 0.01, ***p < 0.001.

Rescue experiments were conducted to explore whether miR-122 mediates the effects of RPPH1 on the cell proliferation, migration, and invasion of HCC cells. HCCLM9 cells were co-transfected with miR-122 inhibitor and shRPPH1 or shNC respectively. The results showed that the miR-122 inhibitor restored the proliferative, migratory, and invasive activities of HCCLM9 cells, which were suppressed by shRPPH1 (Fig. 5A-D). Additionally, the miR-122 inhibitor reversed the downregulation of N-cadherin, Vimentin, and Snail, and decreased the expression of E-cadherin induced by shRPPH1 (Fig. 5E). We demonstrate that miR-122 mediates the effects of RPPH1 on the proliferation, migration, and invasion of HCC cells.

### miR-122-mediated Wnt1/β-catenin signaling is essential for the role of RPPH1 in HCC

**Figure 6:**
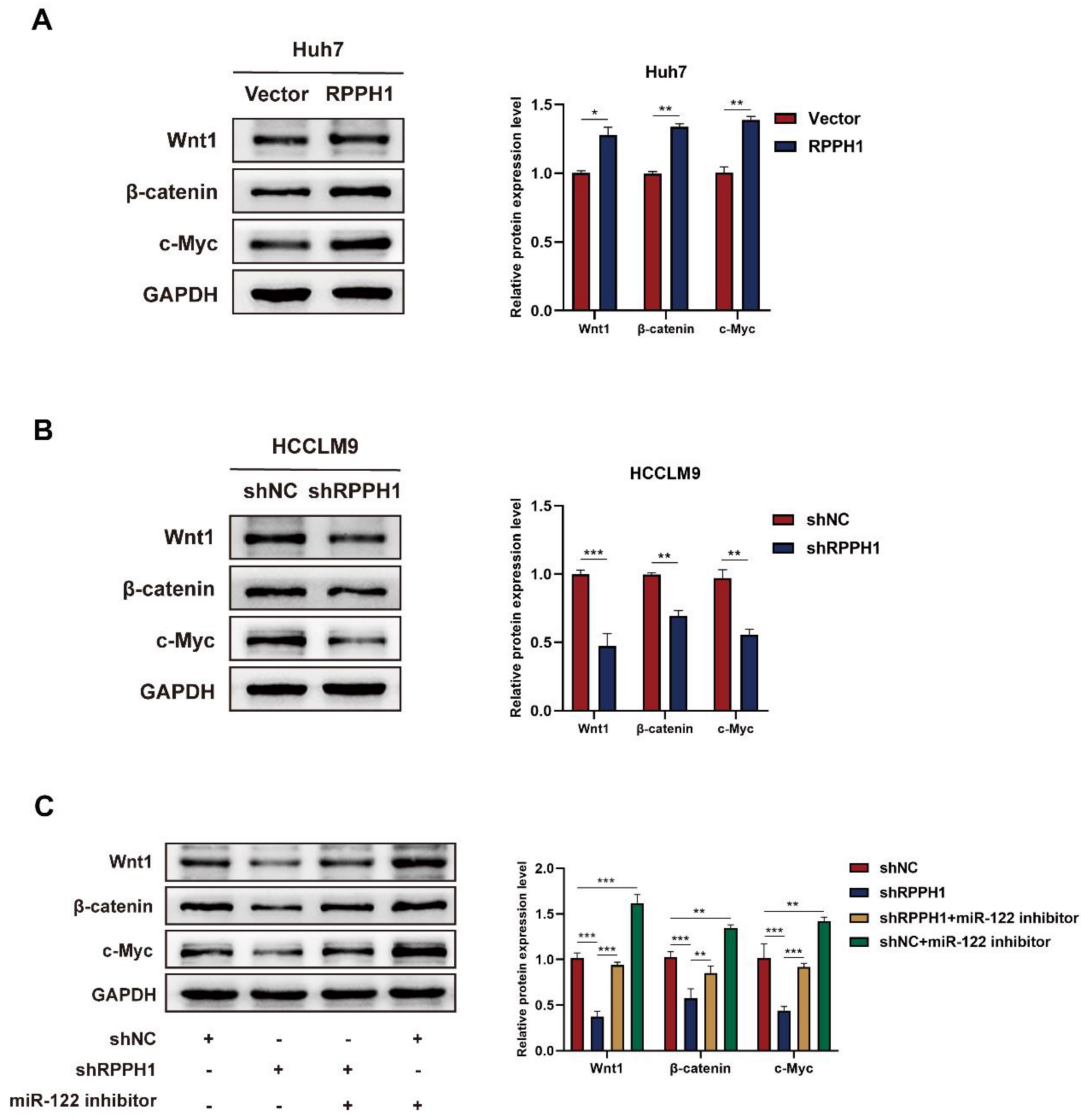
** RPPH1 activates Wnt1/β-catenin signaling by sponging miR-122. (A, B)** Protein levels of Wnt1, β-catenin, and c-Myc after transfection with RPPH1 or shRPPH1 in HCC cells. **(C)** Protein levels of Wnt1, β-catenin, and c-Myc after co-transfection with the miR122 inhibitor and shRPPH1. Data are presented as mean ± SD. *p < 0.05, **p < 0.01, ***p < 0.001.

To evaluate whether Wnt1 is the main factor mediating the biological function of RPPH1 in HCC cells. Western blot assays showed that the protein level of Wnt1, β-catenin, and c-Myc were prominently increased by RPPH1 (Fig. 6A) and reduced by shRPPH1 (Fig. 6B). The co-transfection experiment was designed to investigate whether RPPH1 regulates the expression of Wnt1 expression, as well as those of downstream targets, by sponging miR-122. The results indicate that the shRPPH1-induced decrease in Wnt1, β-catenin, and c-Myc protein expression was remarkably reversed by the miR-122 inhibitor (Fig. 6C), suggesting that miR-122 negatively regulates the expression of Wnt1 and mediates the effect of RPPH1 on Wnt1/β-catenin signaling activity.

## Discussion

Increasing evidence has revealed the biological roles of non-coding RNAs, especially lncRNAs and miRNAs, in tumorigenesis and has provided new strategies for research on the molecular mechanisms of tumor pathogenesis 14-16. In the previous studies, the actions of numerous lncRNAs, such as ubiquitin-conjugating enzyme E2C pseudogene 3 (UBE2CP3) 17, lnc-DILC (lncRNA downregulated in liver cancer stem cells) 18, and cancer susceptibility candidate 2 (CASC2) 19, have been confirmed in hepatocarcinogenesis.

RPPH1 is an RNA subunit of RNase P, which is reported to be abnormally overexpressed in the neocortex of patients with seizures and in tumor tissues of patients with gastrointestinal cancer 20. Previous studies have reported that RPPH1 enhanced CRC cell migration and invasion both *in vitro* and *in vivo*. Mechanistically, RPPH1 binds to TUBB3 to prevent its ubiquitination and then induces EMT in colorectal cancer cells 12. RPPH1 also predicts poor prognosis and regulates cell proliferation and migration by repressing P21 expression in gastric cancer 21. In this study, we examined the expression, function, and underlying mechanism of RPPH1 in HCC. Our results revealed that RPPH1 was significantly increased in HCC tissues and cell lines. Additionally, gain- and loss-of-function experiments were performed to explore the roles of RPPH1 in cell proliferation, apoptosis, migration, and invasion in HCC. As expected, RPPH1 knockdown decreased cell viability, inhibited metastasis, and promoted cell apoptosis. These results indicate that RPPH1 participates in HCC growth. RPPH1 is mainly located in cytoplasm and predominantly expressed in HCC but not in normal liver. However, recent study revealed that RNAs previously considered non-coding, such as a subset of HCC-specific lncRNAs are translated into functional small proteins 22, particularly identification of novel micropeptides derived from HCC-specific lncRNA plays a role in cell proliferations 23. These vital roles and unique properties of lncRNAs suggest that the proteome is more complex than previously anticipated. Future studies will be needed to investigate the role of lncRNAs in hepatocarcinogenesis.

To investigate the potential mechanism of RPPH1 in HCC cells. It has been reported that lncRNAs primarily exert their effects by serving as ceRNAs. We screened the downstream miRNA targets of RPPH1. The bioinformatics results suggested that RPPH1 plays a promoting role in HCC cells by sponging miR-122, which is a liver-specific miRNA that plays an important role in liver development and diseases 24. Previous studies demonstrated that RPPH1 activated Wnt/β-catenin signaling to ameliorate amyloid-β induced neuronal apoptosis in SK-N-SH cells via direct targeting miR-122 25. RPPH1 functions as a tumor promoter and plays an important role in advancing tumorigenesis by targeting miR-122 in breast cancer 26. We demonstrated that miR-122 expression was decreased in tumor tissues and HCC cells compared to normal controls, and the decrease, which was correlated with poor overall survival, had a significant linear correlation with the increase in RPPH1 in HCC. In RPPH1 overexpression and knockdown models, we found that the level of miR-122 was decreased by RPPH1 and increased by shRPPH1. Next, rescue experiments were conducted in co-transfected cells. We found that the addition of the miR-122 inhibitor enhanced cell-proliferation, migration, and invasion abilities that were weakened by shRPPH1. These findings demonstrate that RPPH1 acts as a molecular sponge for miR-122 in HCC cells.

A previous study showed that miR-122 suppresses cell proliferation and induces cell apoptosis in HCC via affecting the Wnt/β-catenin pathway by post-transcriptionally regulating the expression of Wnt1^27^. Numerous studies have shown that the Wnt/β-catenin-mediated signaling cascade plays a crucial role in hepatic oncogenesis 28. Indeed, miR-612 has been shown to suppress the migration and invasion of HCC, partially by HADHA-mediating lipid reprogramming, and to inhibit the formation of invadopodia and Wnt/β-catenin-regulated EMT progression 29. LncRNA LINC00662 activates Wnt/β-catenin signaling in HCC cells and further promotes HCC cell proliferation, cell cycle, and invasion and represses HCC cell apoptosis 30. Thus, we hypothesized that RPPH1 might exert its effect by stimulating the Wnt1/β-catenin pathway by interacting with miR-122. We detected the expression of Wnt1, β-catenin, and c-Myc in RPPH1 overexpression and knockdown models and detected the expression of those proteins in HCCLM9 cells co-transfected with the miR-122 inhibitor and shRPPH1 or shNC. As expected, the results showed that the protein level of Wnt1, β-catenin, and c-Myc were decreased in the RPPH1 knockdown group, and this decrease was reversed by the miR-122 inhibitor.

In summary, our study determined the expression and functions of lncRNA RPPH1 in HCC. More importantly, we showed that RPPH1 supports cell viability and metastasis by activating the Wnt1/β-catenin pathway by serving as a molecular sponge for miR-122 in hepatocellular carcinoma. Further, these findings may have identified a prognostic marker that can be used as an important therapeutic target in the treatment of liver cancer.

## Materials and methods

### Tissue samples

HCC samples and their matched para-carcinoma tissues were collected from 54 surgical patients at the First Affiliated Hospital of Chongqing Medical University. The general features of the patients included in the study are listed in Table 1. All procedures performed in this study involving human participants were in accordance with the Declaration of Helsinki. The study was approved by the Medical Ethics Committee of the First Affiliated Hospital of Chongqing Medical University and informed consent was taken from all the patients.

### Cell culture

Cell lines (LO2, HCCLM9, HCCLM3, SNU-449, SK-Hep1, Hep3B and Huh7) were provided by the Cell Bank of the Type Culture Collection of the Chinese Academy of Sciences (Shanghai, China) or its collaborators. All cells were cultured in Dulbecco's modified Eagle's medium (Gibco, CA, USA) supplemented with 10% fetal bovine serum (FBS, Biological Industries, Israel) in a humidified atmosphere containing 5% CO_2_ at 37°C.

### Cell transfection

The pHB-Scrambled shRNA (shNC) and pHB-RPPH1 shRNA (shRPPH1) plasmids, as well as pcDNA3.1-Control (Vector) and pcDNA3.1-RPPH1 (RPPH1) plasmids were constructed by HanBio (Shanghai, China). The miR-122 inhibitor, mimics, and matching negative controls (NC inhibitor and NC mimics) were purchased from GenePharma (Shanghai, China). The cells were transfected using Lipofectamine 2000 reagent (Invitrogen, CA, USA) according to the manufacturer's protocol.

### Quantitative real‑time polymerase chain reaction

Total RNA was extracted from tissues and cell lines using TRIzol reagent (Invitrogen). For lncRNAs, the SureScript First-Strand cDNA Synthesis Kit (Genecopoeia, Guangzhou, China) was used for reverse transcription. The primer sequences were as follows: RPPH1, 5'-ACTCCACTCCCATGTCCCT-3'(F), and 5′-GGTCCACGGCATCTCCTG-3′(R); miR-122, 5′-GTGACAATGGTGGAATGTGG-3′(F), and 5′-AAAGCAAACGATGCCAAGAC-3′(R); U6, 5′-TCGGCAGCACAT ATACTAAAATTGG-3′(F), and 5'-ACGAATTTGCGTGTCATCCT-3'(R); U3, 5'-TTCTCTGAGCGTGTAGAGCACCGA-3'(F), and 5'-GATCATCAATGGCTGACGGCAGTT-3'(R); and β-actin, 5'-TCCTGTGGCATCCACGAAACT-3'(F), and 5'-GAAGCATTTGCGGTGGACGAT-3'(R). qRT-PCR was performed using BlazeTaq^TM^ SYBR Green qPCR Mix 2.0 (Genecopoeia) and was conducted on the HT7500 System (Applied Biosystems, US) following the unit manual. The relative quantification expression was calculated using the comparative 2^-ΔΔ^Ct method.

### Subcellular RNA fractionation

The PARIS Kit (Invitrogen) was used to isolate cytoplasmic and nuclear RNA fractions, and the samples were subsequently analyzed by qRT-PCR, with β-actin used as the cytoplasmic endogenous control and U3 small nuclear RNA as the nuclear endogenous control. The experiments were conducted according to the instructions.

### MTS (3-4,5-Dimethylthiazol-2-yl)-5-(3-carboxymethoxyphenyl)-2-(4-sulfophenyl)-2H-tetrazolium) assay

The CellTiter 96 AQueous One Solution Cell Proliferation Assay kit (Promega, WI, USA) was used according to the manufacturer's instructions. In brief, HCC cells suspensions were added to a 96-well plate at a density of 2 × 10^4^/well. A total of 20 μL of One Solution reagent was added to each well of the 96-well plate, which contained the samples in 100 µl of culture medium, at the same time every day for 4 days. Then, the plate was incubated at 37°C for 2 h in a humidified 5% CO_2_ atmosphere. Finally, the absorbance at 490 nm was recorded using a Multiskan spectrum (Thermo Fisher Scientific, MA, USA).

### EdU (5-Ethynyl-2'- deoxyuridine incorporation) assay

The EdU Assay Kit (Ribobio, Guangzhou, China) was used according to the manufacturer's instructions. Briefly, 2 × 10^4^ transfected cells per well were seeded into 24-well plates with coverslips. Twenty-four hours later, the culture medium was replaced with medium containing EdU (final concentration, 50 µM) for 2 h at 37°C. Then, the cells were fixed with 4% formaldehyde for 30 min and treated with 0.5% Triton X-100 for 10 min to permeabilize the cells. For cell labeling, the Apollo Reaction Cocktail was used for 30 min. For DNA labeling, Hoechst was applied for 30 min. The images were captured using a fluorescence microscope (Zeiss, Jena, Germany).

### Colony formation assay

Twenty-four hours after transfection, the cells were seeded in a 6-well plate (500 cells per well) and cultured with complete medium for 2 weeks. When visible to the naked eye, the colonies were fixed with 4% paraformaldehyde for 30 min and stained with 0.5% crystal violet for 30 min at room temperature. Finally, the rate of colony formation was calculated.

### Flow cytometry and terminal deoxynucleotidyl transferase mediated dUTP-fluorescein nick end labeling (TUNEL) staining assay

Transfected cells were harvested and stained with Annexin V-FITC/PI Annexin V-FITC/PI Apoptosis Detection Kit (Beijing 4A Biotech Co., Ltd, Beijing, China) according to the manufacturer's instructions. Apoptotic cells were analyzed by flow cytometry (CytoFLEX, Beckman Coulter, USA). For the TUNEL assay, cells transfected with RPPH1 overexpression or knockdown plasmids were plated on 24-well plates with coverslips. Twenty-four hours later, hepatic apoptosis was determined using the YF594 TUNEL Assay Apoptosis Detection Kit (UE Everbright Inc., Suzhou, China) in line with the manufacturer's protocol. DAPI was used to stain the cell nucleus. The cells were observed and photographed under a fluorescence microscope (Zeiss, Jena, Germany) at 400× magnification.

### Wound healing assay

Twenty-four hours after transfection, the cells were seeded into 6-well dishes. When the cell density reached 95%, the monolayers were scratched using a 10 μl pipette tip and washed with phosphate-buffered saline (PBS). Subsequently, the cells were cultured in DMEM without FBS. The cells were photographed at 0 h and 24 h with an inverted microscope (Olympus, Tokyo, Japan) at 200 × magnification. The proportion of wound healing was calculated using ImageJ software (NIH, Bethesda, USA).

### Transwell assay

The 8-μm pore Transwell chamber (Costar, Cambridge, USA) was used to process the transwell assay. Cells were collected and suspended in serum-free medium and placed into the upper compartment coated with (invasion) or without (migration) Matrigel (BD Bioscience, San Jose, CA, USA). Then, the chamber was placed into the cell culture plate containing complete medium and incubated at 37°C for 24 h. The cells inside the upper compartment were removed with cotton swabs. After fixation with 4% paraformaldehyde for 20 min, the migrated cells were stained with 0.5% crystal violet for 30 min. The migrated and invaded cells were finally counted under a fluorescence microscope (Zeiss) at 100× magnification.

### Western blot analysis

HCC cells were lysed with RIPA lysis buffer (Beyotime, Shanghai, China) containing 1% phosphatase inhibitor cocktail and 1% protease inhibitor cocktail (Bimake, Shanghai, China). The protein concentrations were quantified with the BCA Protein Assay Kit (Beyotime). Protein samples were separated by 10% SDS-PAGE and then electroblotted onto PVDF membranes (Millipore, VT, USA). After blocking with 5% skim milk in TBST for 2 h, the membranes were then incubated with the primary antibody overnight at 4°C. Corresponding HRP-conjugated secondary antibodies (Cell Signaling Technology, MA, USA) were used to incubate the membranes for 1 h at room temperature. The protein bands were visualized using the ECL Detection Kit (Advansta, CA, USA). The primary antibodies used in this experiment included anti-Wnt1 antibody (Proteintech Europe, Manchester, UK), anti-β-catenin antibody (Abcam, MA, USA), anti-c-Myc antibody (Abways Technology, Shanghai, China), anti-E-cadherin antibody and anti-N-cadherin antibody (Cell Signaling Technology, Danvers, USA), anti-Vimentin antibody (Bimake, Houston, USA), anti-Snail antibody (Wanleibio, Shenyang, China), and anti-GAPDH antibody (Abways Technology, Shanghai, China).

### Dual-luciferase reporter gene assay

The binding site region of RPPH1 mRNA and miR-122, including the wild-type (wt) RPPH1-3'-UTR and mutant (mut) RPPH1-3'-UTR, was cloned into the pSI-Check2 vector (HanBio). HCCLM9 cells were co-transfected with the vectors and the miR-122 or NC mimics for 48 h. Next, the supernatants were collected, and the luciferase activity was measured using a dual-luciferase reporter assay system (HanBio).

### *In vivo* tumourigenicity

BALB/c nude mice (4 weeks old) were obtained from Beijing HFK Bioscience Co. Ltd. (Beijing, China) and randomly divided into two groups (n = 5). After transfection with shNC and shRPPH1, HCCLM9 cells (2 × 10^6^) were implanted into the right flank of the mice via subcutaneous injection. The tumor volume was monitored weekly for 4 weeks and calculated as follows, volume = (length × width^2^)/2. Finally, all mice were sacrificed under excessive anesthesia. Tumor tissues were harvested and photographed. The animal experiments were approved by the Ethics Committee of Chongqing Medical University.

### In silico analysis

Transcriptome data of four paired TCGA-LIHC and corresponding normal tissues were obtained from GDC Data Portal (portal.gdc.cancer.gov), and 'edgeR' and 'Deseq2' packages were used to obtain differentially expressed genes (DEGs) in the cohort. DEGs with log2-fold change ≥ 1 or ≤ -1 and P-values < 0.05 were selected. Among the selected DEGs, lncRNAs were annotated using ensemble ID from The Atlas of Noncoding RNA In Cancer (TANRIC, www.tanric.org).

### Statistical analysis

SPSS 26.0 software (SPSS Inc., Chicago, USA) and GraphPad Prism 8.0 (GraphPad, CA, USA) were applied to perform data analysis. All data are shown as the mean ± standard deviation (SD). The normality of the quantitative data was examined. The Wilcoxon matched-pairs signed-rank test, Student's *t*-test, and one-way ANOVA were used to perform statistical analysis, as appropriate. *p*-values < 0.05 were considered to indicate statistical significance.

## Figures and Tables

**Table 1:** Basic characteristics of the included patients.

Characteristics	Number of cases n (%)
Gender	
Male	33 (61.1)
Female	21 (38.9)
Age (years)	
≤60	30 (55.6)
>60	24 (44.4)
Tumor size (cm)	
≤5	29 (53.7)
>5	25 (46.3)
AFP	
≤20	36 (66.7)
>20	18 (33.3)
Liver cirrhosis	
Absence	23 (42.6)
Presence	31 (57.4)
Tumor number	
Solitary	32 (59.3)
Multiple (≥2)	22 (40.7)
